# Symptoms of COPD in the absence of airflow obstruction are more indicative of pre-COPD than overdiagnosis

**DOI:** 10.1183/23120541.00264-2024

**Published:** 2024-09-30

**Authors:** Daniella A. Spittle, Maximillian Thomas, Caitlin Stevens, Abdulrhman Gazwani, Sally Fenton, Joshua De Soyza, Alice M. Turner

**Affiliations:** 1 Institute of Inflammation and Ageing, University of Birmingham, Birmingham, UK; 2 University Hospitals Birmingham Foundation Trust, Birmingham, UK; 3 School of Sport, Exercise and Rehabilitation Sciences, University of Birmingham, Birmingham, UK; 4 NIHR Birmingham Biomedical Research Centre, Birmingham, UK; 5 Institute of Applied Health Research, University of Birmingham, Birmingham, UK

## Abstract

**Background:**

Dysfunction of the small airways is a precursor of COPD but is not detectable on standard spirometric testing until significant destruction has occurred. A proportion of COPD patients have a forced expiratory volume in 1 s (FEV_1_)/forced vital capacity (FVC) <0.7 which is greater than the lower limit of normal (LLN), when adjusted for their age and sex. It is not understood whether this group of patients, known as “discordant COPD”, are representative of “early COPD” or overdiagnosis.

**Methods:**

We sought to characterise discordant COPD (disCOPD) using radiology, lung function, serum biomarkers, activity monitoring and quality-of-life scores, comparing with COPD patients with an FEV_1_/FVC <0.7 and <LLN and healthy, age-matched controls.

**Results:**

Six out of eight serum biomarkers were significantly different in the disCOPD group *versus* healthy controls, as were the scores of all four quality-of-life questionnaires. Activity monitoring revealed similar levels of sedentary time between the disCOPD group and concordant COPD (conCOPD). Computed tomography analysis showed less involvement of small airway dysfunction and emphysema in the disCOPD group *versus* conCOPD.

**Conclusions:**

Collectively, our findings support the hypothesis that disCOPD is a clinically relevant phenomenon that represents a pre-COPD state. Identification of such patients is important for early intervention and management before progression to fully established COPD.

## Shareable abstract

**Discordant COPD (FEV_1_/FVC <0.7, >LLN) is a clinically relevant phenomenon that represents an early COPD state, rather than being overdiagnosis of COPD, as demonstrated by CT analysis, serum biomarker levels, activity monitoring and quality of life scores.** https://bit.ly/3WG8wYT

## Introduction

COPD is a progressive respiratory condition that affects nearly 2% of the UK adult population, and causes significant morbidity and mortality [1]. It typically affects older adults, frequently with a history of tobacco smoking, commonly causing dyspnoea, wheeze, a cough that may be productive and frequent exacerbations. Small airway dysfunction (SAD) is a precursor and central feature of COPD. The small airways are known as the “silent” zone, as changes such as airway remodelling, mucus plugging and immune cell infiltration occur in the absence of clinical symptoms [2]. Changes in standard lung function only become detectable once around 75% of small airways have been destroyed [3]. Identifying patients who may have SAD but have not yet progressed to fully established COPD has important implications for intervention and management of these “pre-COPD” patients. The Global Initiative for COPD 2023 report identified these pre-COPD patients as a priority for further research, specifically the identification of patients that later develop COPD and assessment of interventions that may slow or halt this process [4]. While computed tomography (CT) guided measurement of lung volumes has been shown to predict progression to COPD, the feasibility and cost-effectiveness of this is questionable [5, 6].

Diagnosis of COPD is typically based on clinical presentation and traditional spirometry showing airflow obstruction. However, spirometry is a poor tool in the early stages of the disease and can result in unreliable measurements [4]. Additionally, there is increasing evidence of age-related decline in the forced expiratory volume in 1 s (FEV_1_)/forced vital capacity (FVC) ratio that is used to diagnose COPD, in the absence of clinically significant respiratory disease [7]. As such, there is a proportion of “discordant” COPD patients, whose FEV_1_/FVC ratio is <0.7, but not less than the lower limit of normal (LLN) when adjusted for their age and sex. This may lead to overdiagnosis of the healthy elderly population. Another group is recognised, referred to as preserved ratio impaired spirometry (PRISm) who have a preserved ratio (>0.7) but impaired spirometry such as reduced FEV_1_ or FVC. This group represents up to 12% of the population and is independently associated with morbidity and mortality [8]. Investigating discordant and PRISm patients through other methods, such as CT scans, other measures of lung function and biomarkers is crucial to help characterise the early stages of the disease and differentiate early COPD changes which require follow-up from age-related changes. There is currently data on CT changes in PRISm patients, as well increasing evidence on the use of biomarkers in characterising SAD [9, 10].

We conceived this study to investigate if “discordant COPD” was more likely to represent disease or overdiagnosis, by conducting more detailed phenotyping of SAD and emphysema, hypothesising that either of these problems could exist prior to the development of spirometrically defined COPD. By using a mixed methodology of radiology, lung function, biomarkers and quality-of-life (QoL) measurements, we aimed to complete a comprehensive assessment of lung pathology and its impact on these patients.

## Methods

### Study design

We used an observational cohort study design, to assess the degree and nature of SAD in COPD cases and healthy, age-matched controls. The study was ethically approved (IRAS ID: 253739) and all subjects gave informed consent.

### Study population

COPD patients and healthy controls were recruited from a multi-site UK National Health Service (NHS) trust in Birmingham, UK. All patients were over the age of 60 years. Control patients were recruited from previous research databases such as the Healthy Elders cohort [11]. The COPD patients were divided into concordant (FEV_1_/FVC <0.7 and <LLN; conCOPD) and discordant (FEV_1_/FVC <0.7 but >LLN; disCOPD). Importantly this is not the same as PRISM, where FEV_1_/FVC is >0.7. The control patients were confirmed to have no obstruction on their spirometry (FEV_1_/FVC >0.7). Exclusion criteria included inability to understand or speak English, and presence of clinically significant lung disease other than COPD. COPD patients were excluded if they had an exacerbation within the prior 6 weeks. Data on comorbid diseases were collected from the medical record.

### Lung function

Pulmonary function measurements were made during the baseline visit. Spirometry, gas transfer and static lung volumes (Ultima PFT Series, Medical Graphics, UK) and forced oscillatory technique (Resmon Pro, Intermedical, UK) were measured by a clinical scientist in a respiratory physiology laboratory. Lung clearance index (5%) was obtained using a nitrogen washout technique (Ultima PFT Series, Medical Graphics, UK). Global Lung Function Initiative (GLI) (2012) reference equations were used to determine % predicted and LLN. % predicted values account for age and sex, thus had potential to offset any eventual differences in these between groups.

### Symptoms and QoL

All patients completed four health-related QoL questionnaires; the St George's Respiratory Questionnaire (SGRQ) for impact of respiratory disease on health; the SF-36 for a measure of self-reported health status; the Baseline Dyspnoea Index (BDI) for impact of dyspnoea on daily life; and the COPD Assessment Test (CAT) for the impact of COPD symptoms on daily life.

### Physical activity monitoring

All subjects were given a GT3X Actigraph accelerometer (Pensacola, Florida, USA) to wear for 1 week, to provide information about their levels of physical activity. The accelerometer was worn on the right hip, and participants were asked to remove the device for sleeping and water-based activities. The GT3X was set to record accelerations in 10-s epochs, which were subsequently converted into activity counts, and interpreted to determine the frequency, intensity and duration of physical activity.

Data collected by the GT3X was analysed using the Actilife Software (Version 6). For inclusion in the analysis, all participants were required to wear the GT3X for ≥10 h, on ≥4 days. Non-wear time was defined as 60 min of zero counts, with a spike tolerance of 2 min [12]. Time spent sedentary, and in light, moderate and vigorous intensity physical activity, were determined using accelerometer cut-points used in previous research with adults (*i.e.* sedentary time ≥100 counts·min^−1^, light-intensity physical activity 101–2019 counts·min^−1^, moderate-to-vigorous intensity physical activity ≥2020 counts·min^−1^) [13].

### Biomarkers

Blood samples were drawn and centrifuged at 1000×g for 10 min to obtain serum. Serum aliquots were stored at −80°C until further required. Blood biomarkers identified in our previous literature review (CC-16, RAGE, SPD, MMP-8, MMP-9, MMP-12, PAI-1 and CCL-18) were measured using commercially available enzyme-linked immunosorbent assay (ELISA) kits or a Luminex®-based platform [10] (details in supplementary material).

### CT scans

COPD patients had CT scans performed to assess for the presence of emphysema and SAD. The study protocol required inspiratory and expiratory scanning. Non-contrast CT imaging was performed in the caudo-cranial direction while patients were supine, fully inhaled and exhaled, with image acquisition using the CT system (SOMATOM go, Siemens Healthineers, Germany). All scans were reviewed by a radiologist for clinical abnormalities prior to quantitative analysis. Parametric response mapping was performed, according to classification criteria described by Vasilescu *et al*. [14]. For full details of CT protocol, see supplementary material.

### Statistical analysis

Statistical analysis was conducted using R and significance assumed at p<0.05. Descriptive statistics were calculated for patient characteristics, including biomarkers, lung function, CT parameters, symptoms and the amount of time spent doing different activity levels for each of the three groups. Comparison of variables between health, discordant and concordant COPD were performed using ANOVA, Kruskal–Wallis or multiple analysis of variance tests. Categorical data were analysed *via* Fisher's exact test. Data are shown as mean±sd for normally distributed data and median (IQR) for non-normally distributed data.

## Results

64 patients were recruited from December 2019 to October 2022. Significant pauses to recruitment and study assessments occurred during the COVID-19 pandemic. Patient characteristics are shown in table 1. Most clinically diagnosed COPD patients were taking treatment, namely short-acting β-agonist (SABA) alone (17.1%), long-acting muscarinic antagonist (LAMA) or LABA (31.4%), or long-acting β-agonist (LAMA)/LABA/inhaled corticosteroid (28.6%), with >95% of conCOPD patients taking regular treatment. Amongst disCOPD patients, regular treatment was far less universal. Fostair (steroid and LABA) and Trimbow (steroid and LABA/LAMA), inhalers likely to penetrate the small airways, were used in 21% disCOPD and 29% conCOPD. The proportion of patients with clinically significant comorbidities that may affect study outcome measures (*e.g.* cardiac disease, which can impact shortness of breath, and orthopaedic issues, which can impact physical activity) did not differ between disCOPD and conCOPD (51% and 43% respectively; p=0.407). Pulmonary hypertension was not reported in the medical history for any included subject.

**TABLE 1:** Subject characteristics and pulmonary function parameters for healthy controls, discordant COPD (disCOPD) and concordant COPD (conCOPD)

	Healthy controls	disCOPD	conCOPD	p-value
**Demographics**				
** **Patients, n	29	14	21	
** **Age years	72.0±8.5	75.5 (8.5)	66.0 (6.5)	**0.002**
** **Sex (M/F), n	8/21	9/5	9/12	0.07
** **Race, % white	100	85.7	90.4	0.1
** **Body mass index kg·m^−2^	25.9±7.8	28.4 (9.5)	26.20 (5.75)	0.3
** **Smoking status (never/ex/current), n	15/14/0	0/12/2	0/11/10	**<0.001**
** **Pack-years	0.0 (14.3)	35.0 (66.5)	42.5 (40)	**<0.001**
**Pulmonary function measurements**				
** **FEV_1_ % pred	105.1±13.5	84.6±8.5	80.9±13.9	**<0.001**
** **FVC % pred	107.0±14.5	93.9±8.5	108.8±14.7	**0.005**
** **FEV_1_/FVC %	76.4±4.4	68.8±3.0	57.6±6.8	**<0.001**
** **MMEF25–75% pred	107.0±31.7	66.1±13.5	41.8±12.6	**<0.001**
** **TLC % pred	98.4±10.6	97.5±10.3	111.4±13.3	**0.001**
** **FRC % pred	92.1±21.5	94.2±15.6	118.5±27.0	**0.001**
** **RV % pred	90.8±20.0	107.3±18.4	116.6±31.6	**0.005**
** **RV/TLC %	38.9±4.5	43.3±5.7	39.1±5.6	**0.073**
** ***T*_LCO_ % pred	109.6±19.3	93.9±20.6	79.9±14.4	**<0.001**
** ***K*_CO_ % pred	103.8±13.9	92.5±19.4	71.2±14.0	**<0.001**
** **VA % pred	97.3±10.7	100.9±6.6	112.2±11.1	**<0.001**
** **LCI	8.20 (1.68)	9.18 (0.98)	8.11 (1.49)	**0.005**
** **R5Hz % pred	110.2±30.1	140.7±54.9	135.8±42.9	0.08
** **X5Hz % pred	106.3±41.2	177.0±63.2	179.3±120.9	**0.03**
** **R11Hz % pred	113.9±30.5	133.1±52.3	131.6±38.6	0.3
** **R19Hz % pred	103.9±22.8	114.8±39.9	114.5±32.7	0.5
** **R5-19	0.32±0.2	0.78±0.40	0.59±0.74	**0.04**

Data are presented as mean±sd or median (IQR) unless indicated otherwise. ANOVA and Kruskal–Wallis tests were performed appropriately. Bold type for p-values denotes statistical significance. FEV_1_: forced expiratory volume in 1 s; FVC: forced vital capacity; MMEF: maximal mid expiratory flow; TLC: total lung capacity; FRC: functional residual capacity; RV: residual volume; *T*_LCO_: transfer factor of the lung for carbon monoxide; *K*_CO_: carbon monoxide transfer coefficient; VA: alveolar volume; LCI: lung clearance index; R#Hz: resistance measured by forced oscillometry technique; X#Hz: reactance measured by forced oscillometry technique.

### Lung function

Most patients had mild COPD, as demonstrated by FEV_1_ >80% predicted; this was our aim in recruitment, to best identify aspects relevant to early diagnosis. ConCOPD patients were younger (p=0.002) with mean transfer factor of the lung for carbon monoxide (*T*_LCO_) and *K*_CO_ <80% predicted (both p<0.001) and elevated total lung capacity and functional residual capacity (both p=0.001) implying that emphysema and hyperinflation are confined to this group. Established SAD markers were more aberrant in COPD *versus* health, with lower FEV_1_/FVC, lower MMEF25–75, raised X5Hz and raised R5-19 on forced oscillation technique (all p≤0.04). Notably, disCOPD exhibited differences in oscillometry from health, and higher lung clearance index (p=0.005).

### Symptoms and QoL outcomes

Four questionnaires were completed that captured QoL measurements for both respiratory-based outcomes (CAT, SGRQ and BDI) and general health status (SF36), shown in table 2.

**TABLE 2:** Quality-of-life scores

	Healthy controls	disCOPD	conCOPD	p-value
**CAT (total score)**	5 (2–8)	16.5 (10.5–27)	24 (13–30.8)	<0.0001
**SGRQ (subscale scores)**				
Symptoms	3.8 (0–33.5)	50.2 (26–71.5)	72.7 (68.3–87.7)	<0.0001
Activity	11.3 (0–29.9)	47.6 (23.1–73)	66.1 (54.5–94.6)	<0.0001
Impact	0 (0–7.1)	18.8 (11.2–49.1)	40.3 (18.2–67.7)	<0.0001
**BDI (total score)**	11 (9.5–12)	9 (5–9)	5 (3–11)	0.004
**SF36 (subscale scores)**				
Physical functioning	92.5 (85–100)	42.5 (12.5–85)	35 (15–67.5)	<0.0001
Role limitations due to physical health	100 (100–100)	62.5 (0–100)	25 (0–100)	0.01
Role limitations due to emotional problems	100 (100–100)	50 (0–100)	66.7 (0–100)	0.01
Energy/fatigue	85 (70–90)	62.5 (30–76.3)	40 (30–67.5)	<0.0001
Emotional wellbeing	90 (85–96)	80 (56–94)	56 (40–78)	0.0001
Social functioning	100 (78.1–100)	75 (34.4–100)	50 (25–75)	0.001
Pain	95 (71.9–100)	62.5 (30–85)	60 (27.5–85)	0.04
General health	80 (70–88.8)	42.5 (30–60)	35 (20–55)	<0.0001

Data are presented as median (IQR) unless otherwise stated. Groups were compared using the Kruskal–Wallis test. CAT n=49, SGRQ n=52, SF36 n=51, BDI n=39. disCOPD: discordant COPD; conCOPD: concordant COPD; CAT: COPD Assessment Test; SGRQ: St George's Respiratory Questionnaire; BDI: Baseline Dyspnoea Index; SF36: Short Form Healthy Survey with 36 questions.

COPD patients reported worse impact on QoL than the control patients in all questionnaires. A sequential trend was observed across all scores, whereby the median value worsened (higher or lower, depending on individual score interpretation) with disCOPD being worse than healthy, and conCOPD being worse than disCOPD. No significance was observed between the disCOPD and conCOPD groups for any questionnaire.

### Physical activity monitoring

27 of the 64 patients in the study returned activity monitors with successful recordings (healthy controls n=14, disCOPD n=4, conCOPD n=9). This is due to a combination of failure to retrieve the devices, loss to follow-up and device failure. Due to the low numbers in each group, only descriptive analysis could be undertaken.

On average, participants wore the accelerometer for 13.0±0.89 h each day. In all three groups, the majority of accelerometer wear time was spent engaged in sedentary time (healthy control=73%, disCOPD=72%, conCOPD=81%). Lower proportions of time spent in moderate-to-vigorous physical activity were seen in COPD overall (healthy control=5%, disCOPD=3%, conCOPD=2%), but light-intensity physical activity seemed to be maintained by disCOPD patients, compared to healthy controls (healthy control=16%, disCOPD=21%, conCOPD=17%).

### Biomarkers

A total of eight biomarkers were measured in the serum of 55 subjects: 18 healthy controls, 13 with disCOPD and 24 with conCOPD. Six out of eight biomarkers displayed significant difference between the three groups. Both PAI-1 and CCL18 were significantly higher in the serum of disCOPD *versus* healthy controls (p=0.02 and p=0.0006, respectively). PAI-1, CCL18 and MMP-8, -9 and -12 were all significantly higher in the conCOPD compared to the healthy controls (p=0.003, p=0.0009, p=0.0005, p<0.0001 and p=0.01, respectively). CC16 was significantly lower in the conCOPD group *versus* healthy controls (p=0.006). The only significant difference observed between the COPD groups was for MMP-12, where serum concentration was significantly higher in the conCOPD group *versus* disCOPD (p=0.02).

### CT scans

35 patients with COPD were scheduled to have a CT scan of whom 22 patients had the required inspiratory and expiratory CT scan, eight patients had a clinical inspiratory CT scan only, four patients declined a CT scan, and one patient died before CT scanning could be performed. No patients had interstitial disease or significant pulmonary vascular abnormalities on imaging.

**FIGURE 1:**
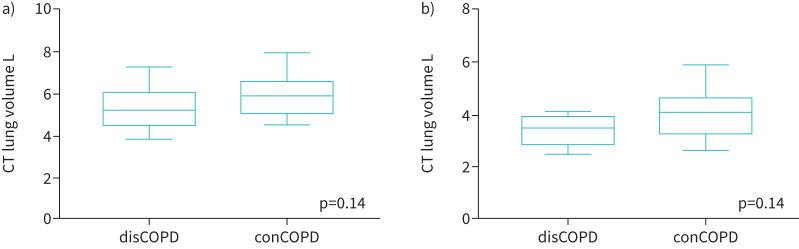
Lung volumes from computed tomography (CT) imaging. Box-and-whisker plots comparing the total pulmonary volume measured in litres in discordant COPD (disCOPD) and concordant COPD (conCOPD) groups using parametric response mapping as a quantitative CT predictive measure. Mann–Whitney tests were performed to compare two individual groups: a) total pulmonary volumes at the peak of inspiration; b) total lung volume throughout the entire lung at the peak of expiration.

**FIGURE 2:**
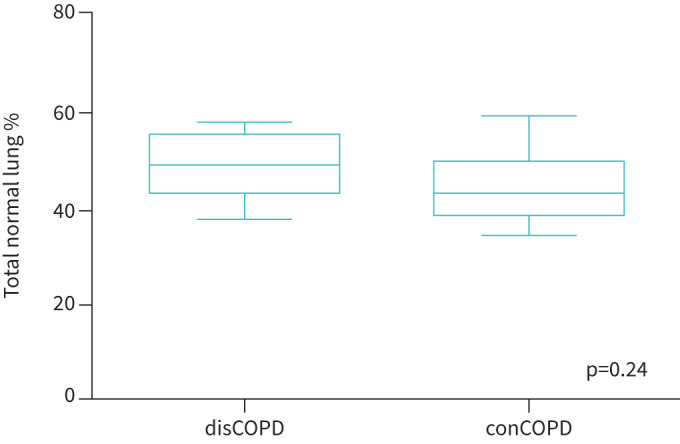
Gas trapping measured by parametric response mapping (PRM). Box-and-whisker plots comparing the proportional normal pulmonary function (PRM Norm) in discordant COPD (disCOPD) and concordant COPD (conCOPD) groups using PRM as a quantitative computed tomography predictive measure. PRM data shown as the percentage of the total lung volume. Mann–Whitney tests were performed to compare two individual groups.

**FIGURE 3:**
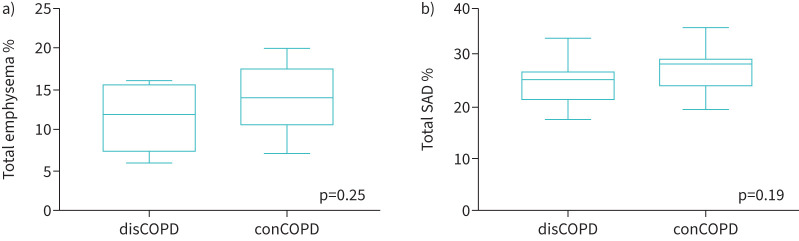
Box-and-whisker plots comparing the proportional emphysematous change and functional small airways disease (SAD) in discordant COPD (disCOPD) and concordant COPD (conCOPD) groups using parametric response mapping as a quantitative computed tomography predictive measure. Mann–Whitney tests were performed to compare two individual groups: a) proportional emphysematous extent change; b) proportional functional SAD.

Quantitative CT measures between discordant and concordant COPD groups were compared (figures 1, 2 and 3). A decrease in total pulmonary volume from inspiration to expiration phase in the disCOPD group was noted (figure 1). Both groups showed a similar range of PRM Norm, with the median value lower in the conCOPD group (figure 2). In addition, we observed a lower proportional PRMEmph and PRMfSAD in disCOPD patients (figure 3). However, the difference in quantitative lung morphology between disCOPD and conCOPD groups was not statistically significant (total pulmonary volume at the peak of inspiration and expiration, p=0.14; PRMNorm, p=0.24; PRMEmph, p=0.25; PRMfSAD, p=0.19). See also supplementary table S1.

## Discussion

These results suggest that disCOPD may be a clinically significant disease, not a form of overdiagnosis. disCOPD patients had symptoms, physical activity, quantitative radiology and biomarker concentrations more closely resembling conCOPD than health. It emphasises the importance of global assessment of the patient, especially when there is high clinical suspicion of COPD, or a COPD-like illness.

Clinical diagnosis of COPD, defined by a diagnostic label having been given to the patient by their primary care provider, did not result in treatment for all disCOPD individuals – prevalence of inhaled treatment was near universal in the case of conCOPD but approaching 50% in the disCOPD group. This may reflect the fact that a COPD diagnosis should be physiologically confirmed, and in the absence of this (in this case FEV_1_/FVC >LLN) arguably those individuals did not meet current guidelines for a COPD diagnosis and thus management. However, the more detailed clinical data suggested a significant symptom burden from all questionnaires, implying that disCOPD patients may require treatment in the same manner as conCOPD.

There was no evidence of emphysema or hyperinflation on lung function in disCOPD patients, despite the presence of SAD, highlighting the potential for misdiagnosis of early COPD due to silent SAD. While formal statistical analysis of accelerometer data was not possible, descriptive estimates suggested similar levels of sedentary time across all groups, with the conCOPD group demonstrating the highest time spent sedentary. There was evidence of some preserved light-intensity physical activity in the disCOPD group when compared to conCOPD patients; there could be potential to further improve levels of light-intensity physical activity if regular inhaled therapy was more common. This again suggests that the disCOPD group are perhaps a precursor to the conCOPD group, and that their physical activity levels may reduce further as the disease progresses. A randomised, placebo-controlled trial investigated the efficacy of inhaled nitrous oxide (iNO) in improving physical activity in pulmonary fibrosis patients [15]. Using actigraphy as a measure of physical activity, the trial showed an increase in moderate-to-vigorous activity in those receiving iNO *versus* placebo.

Studies showing the benefit of inhaled bronchodilators, in the absence of airflow obstruction, have not shown clear benefit for physical activity levels, though evidence is limited [16].

Blood biomarkers were also indicative of a pathophysiological burden in disCOPD. A broad range of serum concentrations were observed in the disCOPD cohort, with a distinct population existing above or below the median value (depending on direction of significance). This population may represent an “at-risk” group of patients that may progress to developing conCOPD. We measured eight biomarkers which have evidence of involvement in SAD [10]. CC16 is expressed largely by club cells in the small airways and is believed to have a protective role within the airways. In a longitudinal cohort, low serum CC16 was associated with accelerated lung function decline [17]. Our results were consistent with this; decreasing levels of serum CC16 from healthy controls to disCOPD, and further in conCOPD, were observed. Similarly, an inverse association with MMP-8 and MMP-9 with lung function was observed. This trend is widely acknowledged in previous COPD studies and is likely reflective of increasing extracellular matrix degradation [18, 19]. More specifically, MMP concentrations have shown a clear association with radiologically confirmed SAD [20]. Serum levels of PAI-1 increased with worsened FEV_1_; PAI-1 is a serine protease inhibitor, associated with several inflammatory conditions, considered to contribute to the pathophysiology of SAD [21, 22]. CCL18 is predominantly secreted by tissue-resident alveolar macrophages and increased serum concentrations are likely proportionate to numbers of the innate immune cells present within the airway [23]. Serving as a pro-inflammatory chemokine, our finding that CCL18 is significantly elevated in the COPD groups is suggestive of increased airway inflammation.

In COPD, CT is the method used for imaging and diagnosing the key morphological changes, including SAD and emphysema [20]. Quantitative approaches help us to overcome subjectivity in sub-classifying the extent and severity of emphysema and gas trapping related more to SAD than emphysema [24, 25, 26]. Vasilescu *et al*. [14] validated the capability of noninvasive PRM CT biomarkers and showed their ability to identify SAD and emphysema morphology. Pompe *et al*. [27] added to the value of using PRM and observed that PRMfSAD and PRMEmph biomarkers were associated with clinical parameters of lung function test, allowing them to identify the presence and severity of COPD. Consistent with our other results, we demonstrated that PRMfSAD and PRMEmph were less impaired in disCOPD than conCOPD patients. This fits with disCOPD being a pre-COPD state, where radiological evidence of disease is present. Disease progression models in COPD suggest that most patients progress from SAD and emphysema to large airway changes over time, rather than the opposite [28].

This study was limited by lower than anticipated recruitment, which is related to the overlap with the COVID-19 pandemic UK peaks. Numbers in this study were lower than in other studies of discordant COPD [29, 30]. Similarly, this limited our ability to appropriately age- and sex-match our cohorts; for lung function and oscillometry measures, we report % predicted values, which account for these confounders. We chose not to stratify by severity of COPD partly for this reason, but also because the distribution of lung function demonstrated that most patients had mild disease (the target population for recruitment to the study). We also only report cross-sectional data, whereas longitudinal studies of spirometry have been able to give evidence of temporal changes [8]. Our ethics committee did not allow CT scanning of healthy controls, although disCOPD patients were still able to be compared with conCOPD.

Despite these absences, a major strength of this study is the breadth of investigations used to investigate our stated aim. Rather than relying on one single measure, we have used symptoms, physiology, radiology and serum biochemistry to holistically assess the clinical relevance of disCOPD, and overall have found a cohesive message: disCOPD patients appear to have similar features to conCOPD, including when compared with healthy controls. This is applicable to the interpretation of the Global Initiative for Chronic Obstructive Lung Disease (GOLD) 2024 report, which advocates the use of a 0.7 FEV_1_/FVC threshold for COPD diagnosis but expresses reservations about overdiagnosis in the elderly, and to UK national guidance, which does not mention the LLN [31, 32]. Our data support the use of the 0.7 threshold in clinical practice, and suggests that, rather than representing overdiagnosis, disCOPD spirometry with symptoms represents disease status.

Use of the 0.7 threshold is likely to make spirometry interpretation easier for the non-specialist and identify COPD at an earlier stage. Identifying the disease at an early stage is likely to have multiple benefits for patients: aside from starting pharmacology earlier, they will be able to access COPD and exacerbation management plans, smoking cessation guidance, physiotherapy where indicated and annual review policies. These approaches all aim to slow progression of the disease, and by accessing them earlier, a greater proportion of lung function may be preserved. Future research could assess asymptomatic patients with this spirometry pattern over time, to see what proportion develop symptoms and FEV_1_/FVC <LLN. Assessing relation of this potential “pre-COPD” state to risk factors for fixed airflow obstruction, such as family history of airways disease, might also be of interest.

### Conclusion

Our data concur with GOLD and suggest that symptomatic patients with FEV_1_/FVC ratio <0.7, but above the LLN, are not overdiagnosed with COPD, but instead represent a clinically significant subset of the disease. When paired with a high clinical suspicion of COPD, such spirometry should trigger holistic investigation, including further lung function and radiology.

## Supplementary material

10.1183/23120541.00264-2024.Supp1 **Please note:** supplementary material is not edited by the Editorial Office, and is uploaded as it has been supplied by the author.
Supplementary material 00264-2024.SUPPLEMENT
