# Supplementary material for: Symptoms of COPD in the absence of airflow obstruction are more indicative of pre-COPD than overdiagnosis
Source: ERJ Open Res. 2024 Sep 30;10(5):00264-2024. doi: 10.1183/23120541.00264-2024 (PMC11440379; doi:10.1183/23120541.00264-2024)
Supplement: Supplementary file 1 [file 00264-2024.SUPPLEMENT.pdf]

Symptoms of COPD in the absence of airflow obstruction are more indicative of pre-COPD than overdiagnosis – supplementary material

Description of commercially-available kits used for biomarker quantification

CC-16 was measured using Human Uteroglobin Quantikine ELISA kit (R&D Systems, Minneapolis, MN, USA); RAGE using Human RAGE Quantikine ELISA kit (R&D Systems); SP-D using Luminex® Discovery Assay (LXSAHM-01) (Bio-Techne Limited, Minneapolis, MN, USA); MMP-8, MMP9 and MMP-12 using Luminex® Human MMP Premixed Kit (Bio-Techne Limited); PAI-1 using Luminex® Human Obesity Premixed Kit (Bio-Techne Limited); CCL18 using Invitrogen PARC/CCL18 Human ELISA kit (Fisher Scientific, Waltham, MA, USA). For biomarkers measured using ELISA, GraphPad Prism 8 version 9.0 (Boston, MS, USA) was used to create standard curves and interpolation of unknown concentrations.

Details of CT scan methodology

Spiral CT images were taken at 120 kVP, mAs automatically modulated, 1mm slice thickness, with a reconstructed Br64f kernel. Scan quality was assessed before quantitative analysis was carried out. Imbio CT Lung Density Analysis™ Software (Imbio, LLC, Minneapolis, MN, v4.1.0) was used for image registration of the paired CT scans and for segmentation of the pulmonary tissue. A voxel-wise map was created with parametric response mapping (PRM), which enabled spatial alignment of inspiratory and expiratory images, thus facilitating regional quantification. Segmentation of lungs and lobes was carried out, with airways excluded from the analysis. During PRM analysis, specific thresholds were set to categorise the voxels. Areas of interest containing features of total pulmonary volume, normal pulmonary function (PRM Norm), functional SAD (PRMfSAD) and emphysema (PRMEmph)

were measured. The classification criteria described by Vasilescu et al. were applied as follows: PRM Norm: inspiration, ≥ -950 Hounsfield Units (HU), expiration, ≥ -856 HU, green; PRMfSAD: inspiration, ≥ -950 HU, expiration, < -856 HU, yellow; PRMEmph: inspiration, < -950 HU, expiration, < -856 HU, red (14). Total, right, left and segmented inspiratory and expiratory pulmonary volumes were recorded.

Step 1: Every patients with COPD participated in dedicated respiratory training sessions.

Step 2: COPD patients underwent CT scans, with careful adjustments made to the CT acquisition parameters.

Step 3: Performed both inspiratory and expiratory scans to evaluate and assess for the presence of emphysema and small airways disease.

Step 4: Image processing used Imbio CT Lung Density Analysis^{TM}
An advanced automated image post-processing algorithm designed to precisely identify and delineate the location and extent of tissue damage. This cutting-edge solution offers both comprehensive visualisation and precise quantification of regions displaying abnormal CT tissue density.

Step 5: Inputs: using functional assessment workflow and parametric response mapping (PRM) processes. During the functional mapping process, the segments are applied to both images (label the lobes of the lungs), followed by registering the inspiration image to the expiration image (enabling a pairwise mapping analysis of the lung images). The images undergo thresholding, followed by the classification of each pair of voxels.

The overlay assigns distinct colour codes to individual lung tissue voxels, facilitating the categorization of tissue into one of three classification groups:

1. Normal Pulmonary Function (PRM Norm):
• Inspiration: ≥ -950 HU,
• Expiration: ≥ -856 HU,
• Colour Code: Green

2. Functional Small Airways Disease (PRMfSAD):
• Inspiration: ≥ -950 HU,
• Expiration: < -856 HU,
• Colour Code: Yellow

3. Emphysematous Change (PRMEmph):
• Inspiration: < -950 HU,
• Expiration: < -856 HU,
• Colour Code: Red

Step 6: Outputs**:** the functional assessment workflow produces two primary outcomes: a functional assessment map and report. This workflow involves the labeling and computation of lung density statistics for the following specific regions within the lung lobes: upper right, middle right, lower right, upper left, and lower left. Total pulmonary volume, right and left lung pulmonary volumes and segmented inspiration and expiratory pulmonary volumes were recorded.

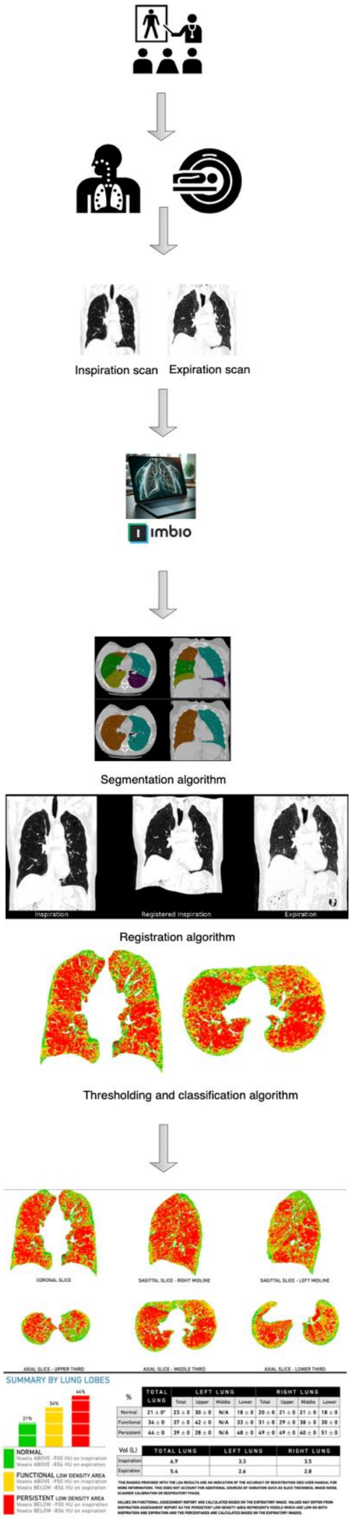

Supplementary Figure 1: A schematic diagram of the Parametric response mapping (PRM) technique process using Imbio computer tomography (CT) Lung Density AnalysisTM. This approach categorises lung attenuation maps by analysing each voxel independently. It achieves this by aligning inspiratory and expiratory images to determine a comprehensive measure of small airway disease and emphysema, including their local distribution and extent. The analysis of the PRM technique consists of the following steps: respiratory training sessions, CT scan acquisition, acquisition of inspiratory and expiratory scanning, image processing, and classification using Imbio software. Image processing started with lung segmentation, followed by registering the inspiration image to the expiration image, thresholding, and finally the classification of each pair of voxels. Then, voxels from attenuation maps can be put into different groups. This lets the clinicians measure and diagnose lung diseases like normal lung (PRM Norm, green), small airway disease (PRMfSAD, yellow), and emphysematous (PRMEmph, red). COPD- Chronic obstructive pulmonary disease.

Supplementary Table 1: Parametric response mapping (PRM) computer tomography (CT)-based quantitative measure for total pulmonary volume measured in litres (L), proportional normal pulmonary function (PRM Norm), proportional emphysematous (PRMEmph) change and functional small airways disease (SAD) (PRMfSAD) in discordant COPD (disCOPD) and concordant COPD (conCOPD) groups using parametric response mapping (PRM) as a quantitative computer tomography (CT) predictive measure. Descriptive statistical analysis. L-left lung; R-right lung; SAD- small airways disease; Emph- emphysematous change; VOL-volume; Insp-inspiration; Exp- expiration; IQR- Interquartile range; COPD- Chronic obstructive pulmonary disease.

CT PRM biomarker	Unit of measurement	disCOPD		conCOPD		Difference	p value
		Median	IQR	Median	IQR		
Total_Normal_Lung	Percentage (%)	49	10	43	10	6	0.2407
Total_Normal_L_Lung	Percentage (%)	51	9	43	12	8	0.1908
Normal_Upper_L_Lung	Percentage (%)	48	9	43	7	5	0.5886
Normal_Middle_L_Lung	Percentage (%)	50	6	40	12	10	0.1134
Normal_Lower_L_Lung	Percentage (%)	55	11	50	15	5	0.1580
Total_Normal_R_Lung	Percentage (%)	50	14	41	9	9	0.3296
Normal_Upper_R_Lung	Percentage (%)	47	16	40	8	7	0.2979
Normal_Middle_R_Lung	Percentage (%)	51	10	41	12	10	0.1796
Normal_Lower_R_Lung	Percentage (%)	52	9	45	14	7	0.3653
SAD_Total_Lung	Percentage (%)	26	5	29	4	-3	0.1896
SAD_Total_L_Lung	Percentage (%)	23	5	27	4	-4	0.2401
SAD_Upper_L_Lung	Percentage (%)	26	7	30	5	-4	0.4190
SAD_Middle_L_Lung	Percentage (%)	23	8	27	6	-4	0.1385
SAD_Lower_L_Lung	Percentage (%)	19	8	23	7	-4	0.4209
SAD_Total_R_Lung	Percentage (%)	25	4	29	6	-4	0.1483
SAD_Upper_R_Lung	Percentage (%)	26	6	31	7	-5	0.1577
SAD_Middle_R_Lung	Percentage (%)	26	5	29	5	-3	0.1048
SAD_Lower_R_Lung	Percentage (%)	21	7	28	7	-7	0.0724
Emph_Total_Lung	Percentage (%)	12	7	14	6	-2	0.2538
Emph_Total_L_Lung	Percentage (%)	12	5	13	7	-1	0.2021
Emph_Upper_L_Lung	Percentage (%)	12	5	14	3	-2	0.6328
Emph_Middle_L_Lung	Percentage (%)	10	4	15	8	-5	0.1042
Emph_Lower_L_Lung	Percentage (%)	8	5	10	8	-2	0.1472
Emph_Total_R_Lung	Percentage (%)	11	7	15	5	-4	0.3825
Emph_Upper_R_Lung	Percentage (%)	12	8	16	5	-4	0.2825
Emph_Middle_R_Lung	Percentage (%)	11	6	15	6	-4	0.2383
Emph_Lower_R_Lung	Percentage (%)	10	3	13	7	-3	0.4196
Total_Lung_VOL_Insp	Litters (L)	5.2	1.5	5.9	1.3	-0.7	0.1390
L_Lung_VOL_Insp	Litters (L)	2.4	0.6	2.8	0.7	-0.4	0.1121
R_Lung_VOL_Insp	Litters (L)	2.7	0.9	3.1	0.7	-0.4	0.2013
Total_Lung_VOL_Exp	Litters (L)	3.5	1.0	4.1	1.2	-0.6	0.1391
L_Lung_VOL_Exp	Litters (L)	1.7	0.6	1.9	0.4	-0.2	0.2140
R_Lung_VOL_Exp	Litters (L)	1.8	0.5	2.1	0.8	-0.3	0.1210
